# Repeated interviews are much better for drug exposure assessment than a single baseline interview

**DOI:** 10.1007/s10654-019-00581-8

**Published:** 2019-11-12

**Authors:** Bruno H. Stricker

**Affiliations:** Department of Epidemiology, Erasmus Medical Center, PO Box 2040, 3000 CA Rotterdam, The Netherlands

In clinical epidemiology, the association between a risk factor and a disease can be flawed by bias and confounding. This should be prevented by a proper design and adjustment. One important aspect is the reliability of exposure and outcome assessment to prevent misclassification bias. Elsewhere in this journal, Cote et al. [1] describe a study on the association between statin use and glioma, in which they employ statin use during interview as the risk factor of interest.

Everybody knows and understands that the strength of a bracelet is determined by the weakest chain. In many pharmaco-epidemiological studies with interview data in the past, exposure assessment was the weakest chain. Why would exposure be more easily misclassified than the outcome in the study of Cote et al.? First of all, due to the fact that the validation of cases was performed by reference to the medical record, false-positive misclassification of the outcome is unlikely, even in the absence of brain pathology. False-negative misclassification is certainly possible but in the end, cases of glioma are probably detected sooner or later and the missing of early cases of such a rare disease in a population-based cohort study, is probably not much of a threat to the risk estimate as long as it is non-differential between users and non-users. However, misclassification of exposure may be a different story. Already in 1977, Copeland et al. [2] demonstrated how devastating exposure, as well as outcome misclassification can be for risk estimates. That a drug interview at baseline as a determinant for events during follow-up leads to exposure misclassification is easily understood. First, because a study on adherence to statins demonstrated that more than 50% of users stops within 2 years [3]. Second, because it is was shown that during a long follow-up period, many non-users of chronic medication according to an interview at baseline will become users because they are started during the years after the baseline interview [4]. Therefore, it is best practice to try to obtain filling data on medicines as such data on continuous use can be analysed with the drug as a time-dependent variable with a lower chance of exposure misclassification [5]. However, several established population-based cohort studies do not have such information, and in most developed countries nowadays privacy legislation makes it difficult to link such studies to health care data from health maintenance organizations and insurance companies. To investigate a duration-effect relationship, Cote et al. tried to circumvent this by using repetitive interview data. Duration of use was estimated by summing use across each 2-year period encompassed by the follow-up questionnaires and classified as never use, 0–4 years, ≥ 4–8 years, and > 8 years [1]. In how far this relates to real use during the whole period could not be verified.

**Fig. 1:**
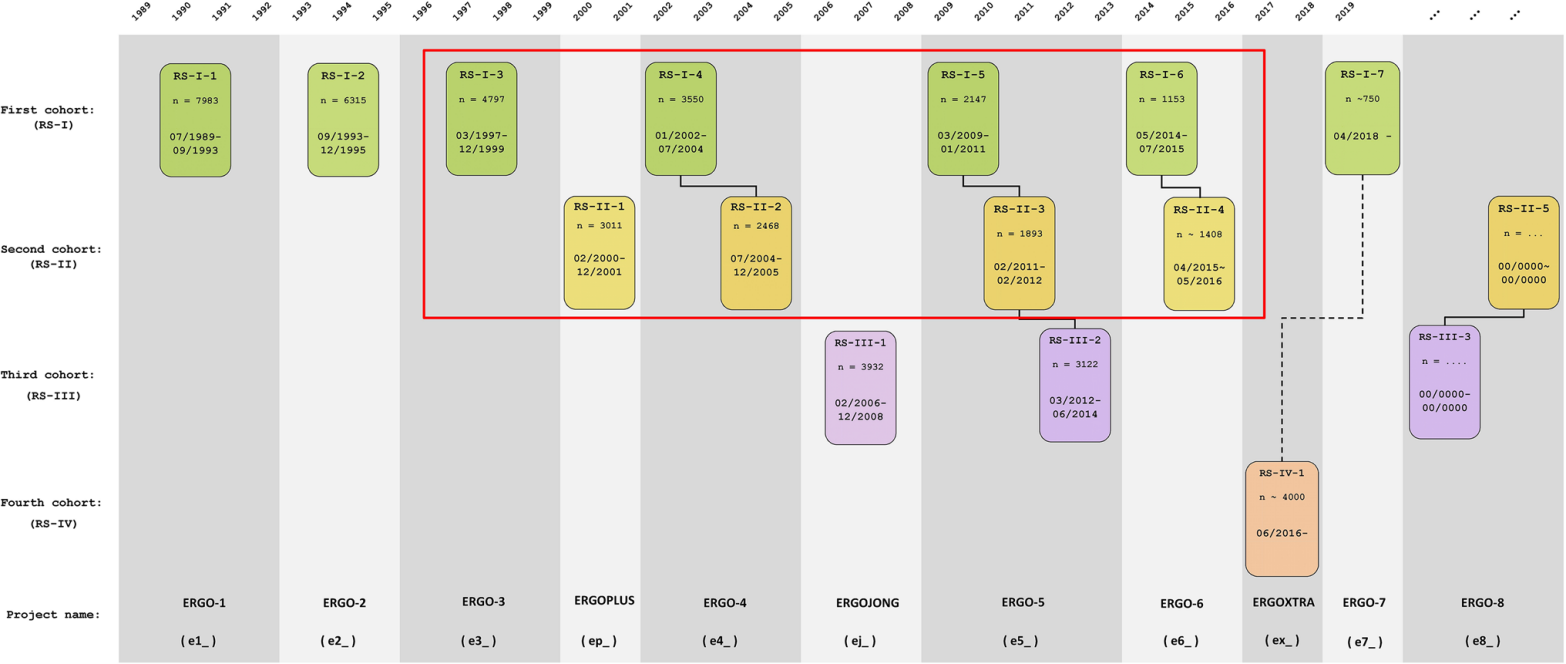
Rotterdam Study cohorts

The author of this Commentary decided to test this in the Rotterdam Study, a population-based prospective cohort study which started in 1990 and of which the details have been described earlier in this journal [6]. To this end, we investigated baseline statin use according to interview in the first two cohorts because the third cohort had less than 4 cycles. Therefore, we studied RSI-3 and RSII-1 during the period 1997–2001 (see Fig. 1), as well as during the subsequent 4 interview cycles that followed until the period 2014–2016. There were 7741 out of 7808 study participants for whom we had a baseline interview during which 906 of them (11.7%) told that they were using statins (confirmed to the interviewers by showing the labelled boxes/canisters). Would this be taken as a proxy indicator of use during follow-up (as is done in many population-based studies with only a baseline medication interview), 129 users according to interview (14.2%) would not have been confirmed by filling data (Tables 1, 2) while no less than 1502 participants classified as non-user would have received statins later during follow-up. This would mean that taking baseline use as an indicator of any use during follow-up would catch only 34% of users according to pharmacy filling data. Comparing these results to any use during follow-up during the successive interviews with pharmacy dispensing data improves the concordance to 84% with only 16% being misclassified as non-users during interview. In Table 3, we show the average number of cumulative days of statin use according to pharmacy filling data within the Rotterdam Study, stratified by the number of interviews during which participants declared that they used statins. Would we take 1 interview as a 2-year period of statin use—as done by Cote et al. because their interviews were biennual—the numbers are remarkably similar to full-time use during the corresponding calendar time but would lead to an overestimation. However, in the Rotterdam Study interviews were taken every 4 years on average and between the 2nd and 3rd interviews there was even a delay of 7 years (Fig. 1). Hence, assuming that statin use would continue between two subsequent interviews in which participants told that they used statins, would overestimate actual use. Although this suggests that pharmacy filling data are a better indicator of actual use, it may be concluded that analyses with repetitive interviews certainly improve the true exposure estimates in comparison to studies where only baseline use is taken as indicator of statin use.

**Table 1:** Users of statins according to interview at baseline and statin users according to pharmacy filling data during follow-up (absolute numbers and percentages of total)

	Use pharmacy	Non-use pharmacy	Total
Use interview	777 (10.0%)	129 (1.7%)	906 (11.7%)
Non-use interview	1502 (19.4%)	5333 (68.9%)	6835 (88.3%)
Total	2279 (70.6%)	5462 (70.6%)	7741 (100%)

Sensitivity 34.1%; specificity 97.6%; kappa 0.38

**Table 2:** Users of statins according to interview during 4 subsequent interviews and statin users according to pharmacy filling data during follow-up (absolute numbers and percentages of total)

	Use pharmacy	Non-use pharmacy	Total
Use interview	1903 (24.6%)	354 (4.6%)	2257 (29.2%)
Non-use interview	376 (4.9%)	5108 (66.0%)	5484 (70.8%)
Total	2279 (29.4%)	5462 (70.6%)	7741 (100%)

Sensitivity 83.5%; specificity 93.5%; kappa 0.77

**Table 3:** Cumulative number of days of use according to pharmacy filling data in comparison to the number of interviews with statin use, assuming continuous use during 2-year and 4-year interview intervals

Number of interviews	Average duration statin use pharmacy	2-Year intervals (days)	%	4-Year intervals	%
1	727 (SE 24)	1–730	99.6	1–1460	49.8
2	1605 (SE 44)	731–1460	110.0	1461–2920	55.0
3	2488 (SE 85)	1461–2190	113.6	2921–4380	56.8
4	3027 (SE 125)	2191–2920	103.7	4381–5840	51.8

Apparently, repeated interview data on drug use are a relatively good proxy indicator for continuous filling data from pharmacies and much better than using a single baseline interview as exposure indicator in a population-base cohort study. Of course, there are a few important considerations to take into account. First, a single example with data from the Rotterdam Study in which repeated interview data on statins are grossly similar to continuous pharmacy data does not prove that we would encounter an identical situation in the dataset of Cote et al. Even stronger, it tells us nothing about the association with glioma, a type of cancer which is too rare for a medium-sized population like the one in Rotterdam. Second, the availability of detailed and prospectively gathered information on type, daily dose, and duration on filled prescriptions facilitates subtle analyses which can never be made with 4 repeated interviews on use of medicines during a more than 8 years-period. But also, we should acknowledge that drug interviews have their strong points because it is probably a better indicator of adherence to therapy and it facilitates information on the use of ‘over-the-counter’ medicines and of medicines obtained during hospital admission which often fail in community pharmacy-derived filling data. Apparently, both interview and pharmacy filling data have their own advantages and limitations, and preferably both types of exposure estimations are used complementary.
